# *Leishmaniavirus* Type 1 Enhances In Vitro Infectivity and Modulates the Immune Response to *Leishmania (Viannia)* Isolates

**DOI:** 10.3390/pathogens14121263

**Published:** 2025-12-10

**Authors:** Armando A. Bonilla Fong, Vanessa J. Pineda, José E. Calzada, Marcia Dalastra Laurenti, Luiz Felipe Domingues Passero, Davis Beltran, Luis Fernando Chaves, Azael Saldaña, Kadir González

**Affiliations:** 1 Programa de Maestría en Ciencias Parasitológicas, Facultad de Ciencias Naturales, Exactas y Tecnología, Universidad de Panamá, Panamá 0816-3366, Panama; aabonillaf22@gmail.com; 2 Departamento de Investigación en Parasitología, Instituto Conmemorativo Gorgas de Estudios de la Salud, Panamá 0816-02593, Panama; vpineda@gorgas.gob.pa (V.J.P.); jcalzada@gorgas.gob.pa (J.E.C.); 3 Facultad de Medicina Veterinaria, Universidad de Panamá, Panamá 0816-3366, Panama; 4 Laboratório de Patologia de Moléstias Infecciosas, Faculdade de Medicina, Universidade de São Paulo, São Paulo 01246-903, SP, Brazil; mdlauren@usp.br; 5 Institute of Biosciences, São Paulo State University (UNESP), São Vicente 11330-900, SP, Brazil; felipe.passero@unesp.br; 6 Institute for Advanced Studies of Ocean, São Paulo State University (UNESP), São Vicente 11350-011, SP, Brazil; 7 Departamento de Investigación en Virología y Biotecnología, Centro de Citometría de Flujo, Instituto Conmemorativo Gorgas de Estudios de la Salud, Panamá 0816-02593, Panama; dbeltran@gorgas.gob.pa; 8 Department of Environmental and Occupational Health, School of Public Health-Bloomington, Bloomington, IN 47408, USA; 9 Department of Geography, Indiana University, Bloomington, IN 47407, USA; 10 Centro de Investigación y Diagnóstico de Enfermedades Parasitarias (CIDEP), Facultad de Medicina, Universidad de Panamá, Panamá 0816-3366, Panama; 11 Departamento de Microbiología Humana, Facultad de Medicina, Universidad de Panamá, Panamá 0816-3366, Panama

**Keywords:** *Leishmaniavirus*, *Leishmania panamensis*, cytokine, immune response, infectivity

## Abstract

*Leishmania RNA virus* 1 (*LRV-1*) is a double-stranded RNA virus identified in several *Leishmania* spp. *LRV-1* has been associated with increased disease severity and therapeutic failure in cutaneous leishmaniasis (CL). Although *LRV-1* has been reported in the Americas, its influence on parasite infectivity and host immune responses remains poorly characterized in Panamanian isolates. In this study, we investigate the in vitro infectivity and immunomodulatory effects of *LRV-1*-positive (*LRV-1*^+^) versus *LRV*-1-negative (*LRV-1*^−^) isolates of *Leishmania (Viannia)*, including clinical strains of *L. (V.) panamensis* and *L. (V.) guyanensis*. A total of 21 isolates (nine *LRV-1*^+^, nine *LRV-1*^−^, and three reference strains) were used to infect human U937 macrophages. The infectivity index (II) was measured at 24, 48, and 72 h post-infection. Cytokine levels of TNF-α, IFN-γ, IL-4, IL-6, IL-10, and IL-17 were quantified by flow cytometry, and IL-1β by ELISA at 24 and 48 h. *LRV-1*^+^ isolates exhibited significantly higher infectivity at 48 h (mean II = 1386.2) and 72 h (mean II = 1316.8) compared to *LRV-1*^−^ isolates (mean II = 714.4 and 571.0, respectively; *p* < 0.001). Two *L. (V.) panamensis LRV-1*^+^ isolates associated with complicated CL cases displayed the highest II values. Cytokine analysis revealed that *LRV-1*^+^ isolates induced elevated TNF-α (*p* < 0.01) and IL-1β (*p* < 0.001), along with reduced IFN-γ (*p* < 0.01), while no significant differences were observed for IL-4, IL-6, IL-10, or IL-17. These findings indicate that *LRV-1* enhances parasite infectivity and promotes a pro-inflammatory cytokine profile, which may contribute to disease persistence and treatment failure.

## 1. Introduction

*Leishmania RNA virus* (*LRV*) is a non-enveloped, double-stranded RNA virus belonging to the family Totiviridae, known to infect protozoan parasites of the genus *Leishmania* in both the Old and New World. Of the four identified species (*LRV-1* to *LRV-4*), only *LRV-1* and *LRV-2* have demonstrated medical relevance due to their presence in *Leishmania* species pathogenic to humans [1,2,3,4,5,6,7]. *LRV-1* is primarily found in American *Leishmania* species of the *Viannia* and *Leishmania* subgenera, while *LRV-2* has been identified in Old World species belonging to the *Leishmania* and *Sauroleishmania* subgenera [8,9,10,11,12,13]. First detected in *L. (V.) guyanensis*, *LRV-1* has been increasingly associated with exacerbated clinical outcomes in cutaneous leishmaniasis (CL), including lesion reactivation, treatment failure, and mucosal involvement [5,14,15,16,17,18].

The host immune response to *Leishmania* is largely governed by macrophages that play a crucial role in controlling *Leishmania* infection. Two main macrophage populations have been described: Th1-induced M1 macrophages (classically activated macrophages), which exhibit a pro-inflammatory profile and can eliminate the parasite by producing reactive oxygen species (ROS), and Th2-induced M2 macrophages (alternatively activated macrophages), which display an anti-inflammatory profile and increase arginase activity, providing a favorable environment for *Leishmania* replication [19,20,21,22].

A critical feature in the pathogenesis of *LRV*-infected *Leishmania* is the virus-driven dysregulation of these immune pathways. An imbalance between pro- and anti-inflammatory cytokines facilitates parasite dissemination and persistence, exacerbating tissue damage [23,24,25]. In *L. (V.) guyanensis*, *LRV-1* has been shown to inhibit the NLRP3 inflammasome, a key innate immune sensor that restricts *Leishmania* replication within macrophages [14,26], while simultaneously upregulating pro-inflammatory mediators such as tumor necrosis factor-alpha (TNF-α), interleukin-6 (IL-6), interleukin-17 (IL-17), and interferon-gamma (IFN-γ) [23,25]; and thereby promoting the persistence of the parasite by improving survival in macrophages [27].

Similar immunomodulatory effects have been described in *L. (V.) braziliensis*, where *LRV-1* infection correlates with increased in vitro infectivity and a cytokine profile skewed toward Th2 responses, impairing parasite control [28,29]. Transcriptomic analyses of infected human macrophages revealed that *LRV-1*-positive *L. (V.) braziliensis* triggers upregulation of type I IFN signaling pathways and pattern recognition receptors, including 2′-5′-oligoadenylate synthetase/ribonuclease L (OAS/RNase L) and retinoic acid-inducible gene I (RIG-I) [30].

Geographically, *LRV-1* has been reported in multiple South American countries, including French Guiana, Suriname, Bolivia, Brazil, Peru, Ecuador, Colombia, and Venezuela, infecting various *Viannia* species such as *L. (V.) guyanensis*, *L. (V.) braziliensis*, *L. (V.) panamensis*, *L. (V.) shawi*, *L. (V.) lainsoni*, *L. (V.) naiffi*, *L. (V.) peruviana*, and *L. (L.) amazonensis* [9,16,30,31,32,33,34,35,36]. In Central America, *LRV-1* has been detected in Costa Rica and Panama, predominantly in *L. (V.) panamensis* [29,36,37].

According to the 2024 PAHO report, Panama remains endemic for CL, recording approximately 1000 new cases annually. *L. (V.) panamensis* is the principal etiological agent, responsible for ~95% of localized cutaneous leishmaniasis (LCL) cases [38,39]. Less frequent but clinically relevant presentations include mucocutaneous leishmaniasis (MCL), disseminated CL (DCL), and anergic diffuse CL (ADCL), also attributed to *L. (V.) panamensis* [40,41,42].

Despite its clinical relevance, studies investigating the presence and pathogenic role of *LRV-1* in *L. (V.) panamensis* remain limited. Preliminary data suggest that, unlike in *L. (V.) guyanensis* or *L. (V.) braziliensis*, *LRV-1* in *L. (V.) panamensis* may not modulate cytokine responses significantly [29]. However, this observation warrants further research, particularly considering emerging reports linking *LRV-1* to severe or treatment-refractory CL caused by *L. (V.) panamensis* [17,43].

Given this context, the present study investigates the role of *LRV-1* in modulating parasite infectivity and immune response in macrophages using characterized *L. (V.) panamensis* and *L. (V.) guyanensis* clinical isolates. By comparing *LRV*-1-positive and negative isolates in an in vitro U937 macrophage infection model, we aim to elucidate potential immunological mechanisms and assess the contribution of *LRV-1* in parasite spreading and persistence. These findings may offer new insights into the role of virus–parasite interactions in the pathogenesis of *Leishmania (Viannia)* infections and support further investigation of *LRV-1* as a potential prognostic biomarker or therapeutic target in endemic regions.

## 2. Materials and Methods

### 2.1. Leishmania Isolates and Cell Lines

Twenty-one isolates of *Leishmania (Viannia)* spp. were analyzed in this study. Eighteen clinical isolates were obtained from patients diagnosed with localized cutaneous leishmaniasis (LCL) in Panama and selected from the biobank of the Department of Parasitology Research at the Instituto Conmemorativo Gorgas de Estudios de la Salud (DIP-ICGES). These isolates had been cryopreserved between 2016 and 2019. The panel included nine *LRV-1* positive isolates (*LRV-1*^+^), nine *LRV-1* negative isolates (*LRV-1*^−^), and three reference strains (Table 1).

Within both the *LRV-1*^+^ and *LRV-1*^−^ groups, eight isolates were identified as *L. (V.) panamensis* and one as *L. (V.) guyanensis*. Most clinical isolates were obtained from patients with uncomplicated LCL. However, two isolates corresponded to cases with atypical clinical features: one from a patient with 11 active lesions, and another from a case showing failure to respond to three cycles of pentavalent antimonial treatment.

For in vitro infection experiments, the human U937 macrophage cell line was employed, acquired from the American Type Culture Collection (ATCC, CRL-1593.2, Manassas, VA, USA).

### 2.2. Ethical Considerations

This study was approved by the Institutional Bioethics Committee of the ICGES (protocol code: 267/CBI/ICGES/21). The research involved retrospective analysis of parasite isolates and did not involve studies in animals or human subjects.

### 2.3. Selection and Characterization of Leishmania Isolates

To compare infectivity and cytokine response, two groups were chosen: Group 1, with nine *LRV-1^+^* isolates, and Group 2, with nine *LRV-1*^−^ isolates (Table 1). Each group included one isolate of *L. (V.) guyanensis* and eight of *L. (V.) panamensis*. The reference strains included *L. (V.) guyanensis LRV-1*^+^ (MHOM/BR/1975/WR4147), *L. (V.) panamensis LRV-1*^−^ (MHOM/PA/1971/LS94), and *L. (V.) braziliensis LRV-1*^−^ (MHOM/BR/1975/M2903). The presence of *LRV-1* had been previously confirmed by RT-PCR targeting a 240 bp fragment from the ORF1 region [36]. Parasite species were confirmed via PCR-RFLP of the *Hsp-70* gene [44].

### 2.4. RNA Extraction and LRV-1 Detection

Parasites were thawed and cultured in Schneider’s insect medium (Gibco™, 11590576, Paisley, United Kingdom) supplemented with 25% heat-inactivated Fetal Bovine Serum (FBS, Gibco™, A56697-01, Grand Island, NY, USA) until the stationary phase. Total RNA was extracted using the RNeasy Mini Kit (QIAGEN, Hilden, Germany) and quantified with a NanoDrop 2000 spectrophotometer (Thermo Fisher Scientific, Wilmington, DE, USA). Reverse transcription was performed using the SuperScript™ IV First-Strand Synthesis SuperMix kit (Thermo Fisher Scientific, 18091050, Vilnius, Lithuania). *LRV-1* detection was carried out by RT-PCR using primers LRV-1F (5′-ATGCCTAAGAGTTTGGATTCG-3′) and LRV-1R (5′-ACAACCAGACGATTGCTGTG-3′) at 0.2 mM of each primer [37]. Amplicons were visualized in 1% agarose gels stained with SYBR^TM^ Safe DNA Gel Stain (Invitrogen, S33102, Carlsbad, CA, USA).

### 2.5. Macrophage Differentiation

U937 cells were cultured in RPMI-1640 (Gibco™, 21875034, Grand Island, NY, USA) supplemented with 10% FBS and differentiated into macrophages using 100 ng/mL Phorbol 12-myristate 13-acetate (PMA; Abcam, ab120297, Waltham, MA, USA) for 72 h as described by Kariyawasam et al. (2017) [29].

### 2.6. In Vitro Infection Assay

Differentiated U937 macrophages (5 × 10^5^ cells/well) were seeded in 24-well plates containing 15 mm round coverslips and infected with stationary-phase promastigotes (day 6 of culture, 1 × 10^7^ parasites/mL). Parasites and cell viability were determined by the Trypan blue exclusion test (Trypan Blue stain, Gibco, 15250-061, Grand Island, NY, USA), and the inoculum was subsequently adjusted to 5 × 10^6^ parasites/well, corresponding to a multiplicity of infection (MOI) of 20:1 (parasite per macrophage). After 3 h, non-internalized parasites were removed. Infections were carried out in triplicate per isolate and repeated in two independent experiments (*n* = 6 per condition). The cultures were maintained in RPMI-1640 medium with 15% FBS at 35 °C and 5% CO_2_. Negative infection controls included uninfected U937 cells, while positive cytokine stimulation controls included U937 cells treated with 0.1 μg/mL *E. coli* O111:B4 lipopolysaccharide (LPS; Sigma-Aldrich, L2630, Saint Louis, MO, USA). At 24, 48, and 72 h post-infection, supernatants were collected and stored at −80 °C for cytokine analysis. Coverslips were washed with PBS, stained using Easy III Stain Kit (Azer Scientific, ES902-16, Morgantown, PA, USA), and mounted with Permount™ (Fisher Chemical, SP15-500, Fair Lawn, NJ, USA) for microscopic analysis. The infection index (II) was calculated as the percentage of infected macrophages multiplied by the average number of amastigotes per cell [29,45].

### 2.7. Cytokine Quantification

Cytokines IL-4, IL-6, IL-10, IL-17, TNF-α, and IFN-γ were quantified in supernatants collected at 24 and 48 h using the BD™ CBA Human Th1/Th2/Th17 Cytokine Kit (Becton Dickinson, 560484, San Diego, CA, USA), following the manufacturer’s protocol. Samples were analyzed in triplicate. Data acquisition was performed with a BD LSRFortessa™ X-20 flow cytometer (BD Biosciences, San Jose, CA, USA) and analyzed using BD FlowJo™ v10.

IL-1β levels were measured using a commercial ELISA kit (Abcam, ab100562, Waltham, MA, USA), according to the manufacturer’s instructions. Samples were analyzed in triplicate using a Multiskan™ FC microplate reader (Thermo Scientific™, Waltham, MA, USA).

### 2.8. Statistical Analysis

Infection indices (II) at 24, 48, and 72 h were compared using two-way ANOVA in GraphPad Prism 8.0.1 (GraphPad Software, Inc., 1995–2024). Cytokine concentrations were analyzed using the Shapiro–Wilk normality test and two-way ANOVA. Associations between infectivity and cytokine expression profiles were assessed using Spearman’s rank correlation. Principal Component Analysis (PCA) using the standardize method, selecting PCs based on eigenvalues greater than 1.0 (the “Kaiser rule”), was assessed. The eigenvalues were computed on a correlation matrix. Both analyses were implemented in GraphPad Prism 8.0.1.

## 3. Results

### 3.1. Infectivity Index (II) of Leishmania (Viannia) Isolates According to LRV-1 Status

**Figure 1:**
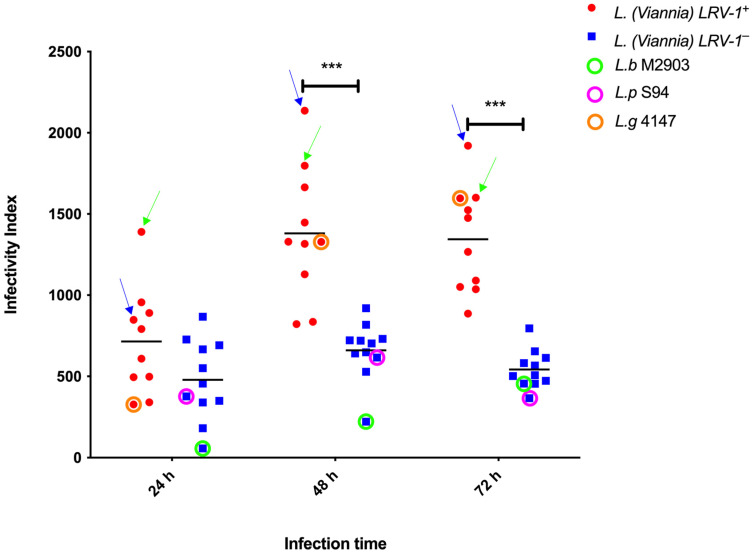
Scatter plot showing differences in infectivity between *Leishmania LRV-1*^+^ (red) and *LRV-1*^−^ (blue) isolates at 24, 48, and 72 h post-infection. The *LRV-1*^+^ group includes nine clinical isolates, as well as the *L. (V.) guyanensis* reference strain 4147 (orange circle). The *LRV-1*^−^ group includes nine clinical isolates, along with the reference strains *L. (V.) panamensis* (LpS94) (pink circle) and *L. (V.) braziliensis* (LbM2903) (green circle). Two *L. (V.) panamensis* isolates with the highest II values are highlighted: blue arrow indicates isolate Lp004; green arrow indicates isolate Lp005. ***: *p* < 0.0001. Tukey’s test was used for multiple comparison correction.

Isolates harboring *LRV-1* (*LRV-1*^+^) demonstrated a significantly higher average infectivity index (II) compared *to LRV-1*-negative (*LRV-1*^−^) isolates (Figure 1). Within the *LRV-1*^+^ group, the II increased markedly from 24 h (mean II = 757.45) to 48 h post-infection (mean II = 1386.23; *p* < 0.001) and remained elevated at 72 h (mean II = 1316.77; *p* < 0.001). In contrast, *LRV-1*^−^ isolates showed no statistically significant variation in II across the three time points (24 h: mean II = 456.00; 48 h: mean II = 714.43; 72 h: mean II = 571.01; *p* > 0.05).

When analyzing *LRV-1*^+^ isolates for the association between their presence and clinical complications, two *L. (V.) panamensis* isolates stood out with the highest II scores: Lp004 (mean II = 1515.30), obtained from a patient with 11 cutaneous lesions, and Lp005 (mean II = 1587.31), isolated from a case requiring three treatment cycles with pentavalent antimonial (Figure 1).

### 3.2. Infectivity Index (II) of Leishmania (Viannia) Isolates According to Species Stratification

**Figure 2:**
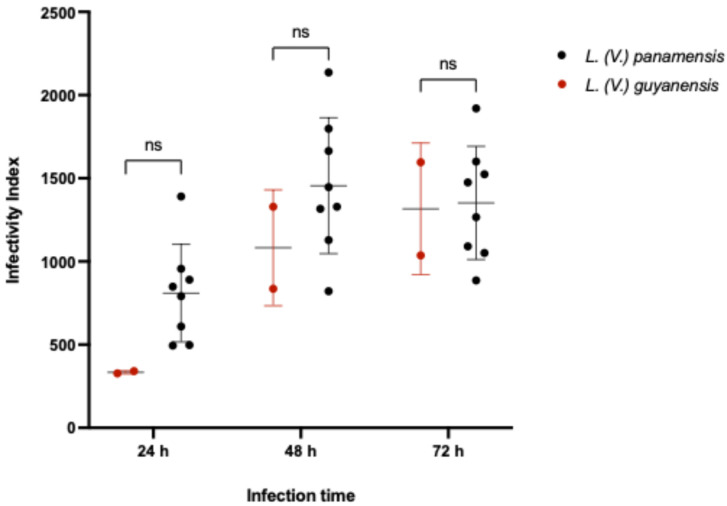
Scatter plot showing differences in the infectivity index (II) between *LRV-1*-positive *L. (V.) panamensis* (black) and *L. (V.) guyanensis* (red) isolates. The panel includes nine clinical isolates from both species, along with the reference strain *L. (V.) guyanensis* 4147. ns: not significant. Two-stage linear step-up procedure of Benjamini, Krieger and Yekutieli were used to multiple test correction.

When infectivity was analyzed at the species level within the *LRV-1*^+^ group, no statistically significant difference was observed between *L. (V.) panamensis* and *L. (V.) guyanensis* isolates using a non-parametric Mann–Whitney test (Figure 2).

**Figure 3:**
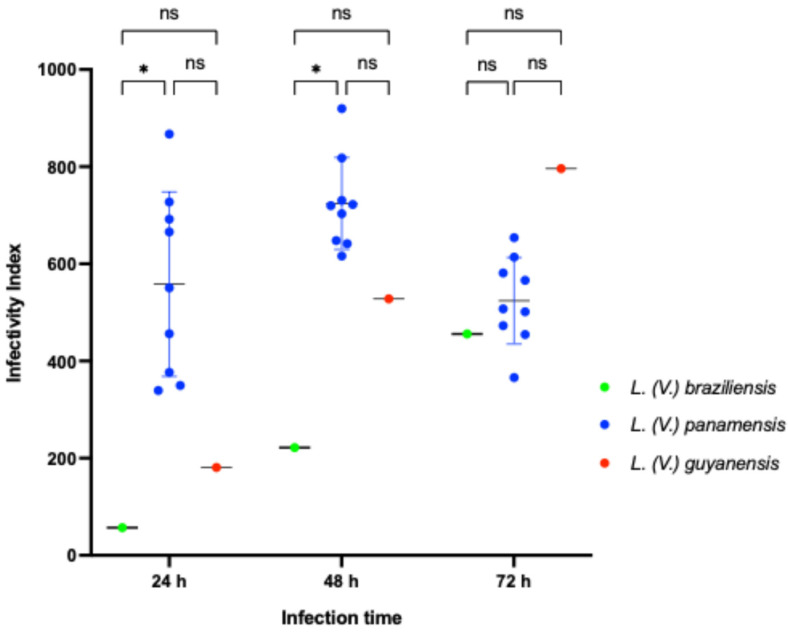
Scatter plot showing differences in the infectivity index (II) among *LRV-1* negative *Leishmania (Viannia)* species. Blue circles represent clinical isolates and the reference strain of *L. (V.) panamensis*; red circle represents the *L. (V.) guyanensis* isolate; green circle corresponds to the reference strain of *L. (V.) braziliensis* (LbM2903). ns: not significant; *: *p* < 0.01. Šídák’s test with a single pooled variance was used for multiple comparisons correction.

Although only one data point was analyzed for the reference strains of *L. (V.) braziliensis* M2903 and *L. (V.) guyanensis LRV-1*^−^ isolate, the infectivity index (II) was compared among the *LRV-1*^−^ isolates. According to the ANOVA, the II varied significantly across species (Figure 3). The *L. (V.) panamensis* isolates showed higher infectivity at 24 and 48 h compared to the reference strain *L. (V.) braziliensis* M2903 (*p* < 0.05). While *L. (V.) guyanensis LRV-1*^−^ isolate and *L. (V.) braziliensis* M2903 showed a trend toward increasing infectivity over time, *L. (V.) panamensis* reached peak infectivity at 48 h, then declined slightly at 72 h.

### 3.3. Modulation of Pro-Inflammatory Cytokines TNF-α, IL-1β, and IFN-γ by LRV-1

**Figure 4:**
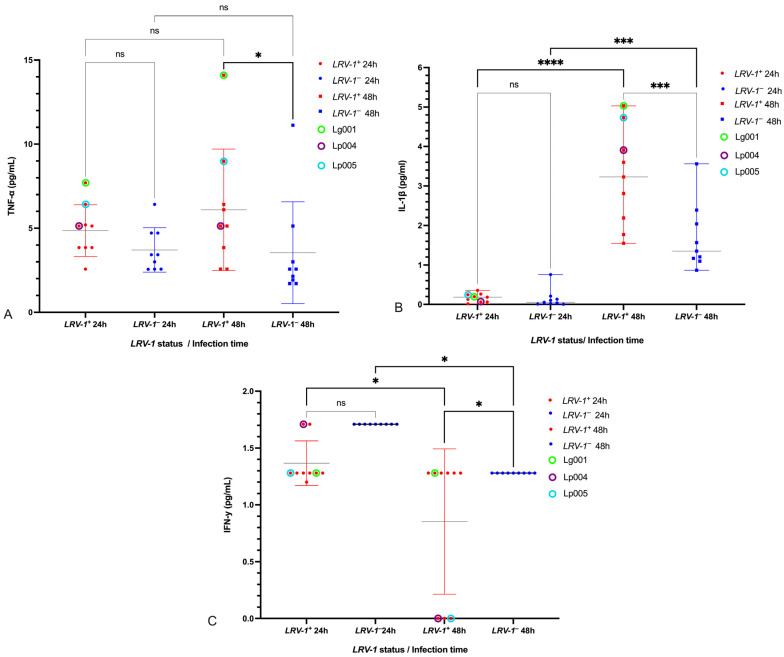
Scatter plots showing the concentrations of TNF-α (**A**), IL-1β (**B**), and IFN-γ (**C**) in the supernatants of U937 cells infected with *Leishmania (Viannia) LRV-1*^+^ and *LRV-1*^−^ isolates at 24 and 48 h post-infection. Statistical comparisons were performed between groups. ns: not significant; *: *p* < 0.01; ***: *p* < 0.0001; ****: *p* < 0.00001. Tukey’s test was used for multiple comparison correction.

At 48 h post-infection, the supernatant of U937 macrophages infected with *LRV-1*^+^ isolates contained significantly higher concentrations of TNF-α (*p* < 0.01) and IL-1β (*p* < 0.0001), but a lower concentration of IFN-γ (*p* < 0.01), compared to macrophages infected with *LRV-1*^−^ isolates (Figure 4, Figures S1 and S2).

Notably, isolates Lg001 and Lp005 induced high TNF-α levels at both 24 and 48 h, while IL-1β concentrations were highest in Lg001, Lp004, and Lp005 at 48 h. IFN-γ levels were undetectable at 48 h for Lp004 and Lp005 (0.00 pg/mL) (Figure 4).

When analyzed by *Leishmania* species, no overall differences in TNF-α or IL-1β levels were found between *LRV-1*^+^ and *LRV-1*^−^ groups (*p* > 0.05).

### 3.4. Cytokines Unaffected by LRV-1 Status

**Figure 5:**
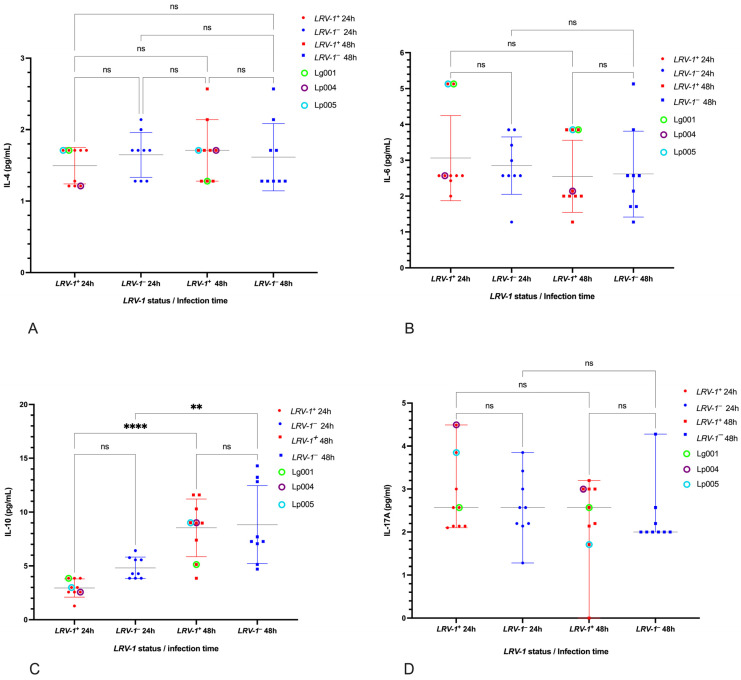
Scatter plots showing the concentrations of IL-4 (**A**), IL-6 (**B**), IL-10 (**C**), and IL-17 (**D**) in the supernatants of U937 cells infected with *Leishmania (Viannia) LRV-1*^+^ and *LRV-1*^−^ isolates at 24 and 48 h post-infection. ns: not significant; **: *p* < 0.001; ****: *p* < 0.00001. Tukey’s test was used for multiple comparison correction.

No statistically significant differences were found in IL-4, IL-6, IL-10, or IL-17 concentrations between *LRV-1*^+^ and *LRV*-1^−^ isolates at 24 or 48 h (*p* > 0.05). However, a general increase in IL-10 was observed at 48 h in both groups (*LRV-1*^+^: *p* < 0.0001; *LRV-1*^−^: *p* < 0.001). No differences were noted among *Leishmania* species for these cytokines (Figure 5).

### 3.5. Cytokine Responses in Reference Strains and LPS Control

At 24 h, the Lg4147 reference strain (*LRV-1*^+^) induced a significantly higher TNF-α concentration (10.30 pg/mL) than the average values for both *LRV-1*^+^ (5.13 pg/mL; *p* < 0.01) and *LRV-1*^−^ (3.42 pg/mL; *p* < 0.001) clinical isolates, as well as the LpS94 strain (3.00 pg/mL; *p* < 0.01) (Figure S3A). IL-1β concentrations were also significantly elevated in Lg4147 and LbM2903 at 24 h compared to clinical isolates (*p* < 0.01 and *p* < 0.001, respectively), although the LbM2903 response declined at 48 h (Figure S3). The LPS control induced robust increases in TNF-α and IL-1β at both time points compared to all parasite isolates (*p* < 0.0001 and *p* < 0.01, respectively. Also, LPS induced an increase in IL-6 at 48 h compared to all clinical isolates (*p* < 0.001) and reference strains (*p* < 0.01). No significant differences in IFN-γ, IL-4, IL-10, and IL-17 were observed between reference strains, clinical isolates, or the LPS control (*p* > 0.05) (Figure S3).

### 3.6. Multivariate and Correlation Analysis

Spearman correlation analysis among *LRV-1*^+^ isolates at 24 h revealed positive correlations between IL-6 and TNF-α (r = 0.65, *p* < 0.03) and between IL-6 and IL-17 (r = 0.60, *p* < 0.04). At 48 h, a positive correlation between IL-6 and TNF-α persisted in this group (r = 0.79, *p* < 0.01). Among *LRV-1*^−^ isolates, a significant negative correlation was found between the II and TNF-α levels at 24 h (r = −0.73, *p* < 0.01). (Figure S4).

**Figure 6:**
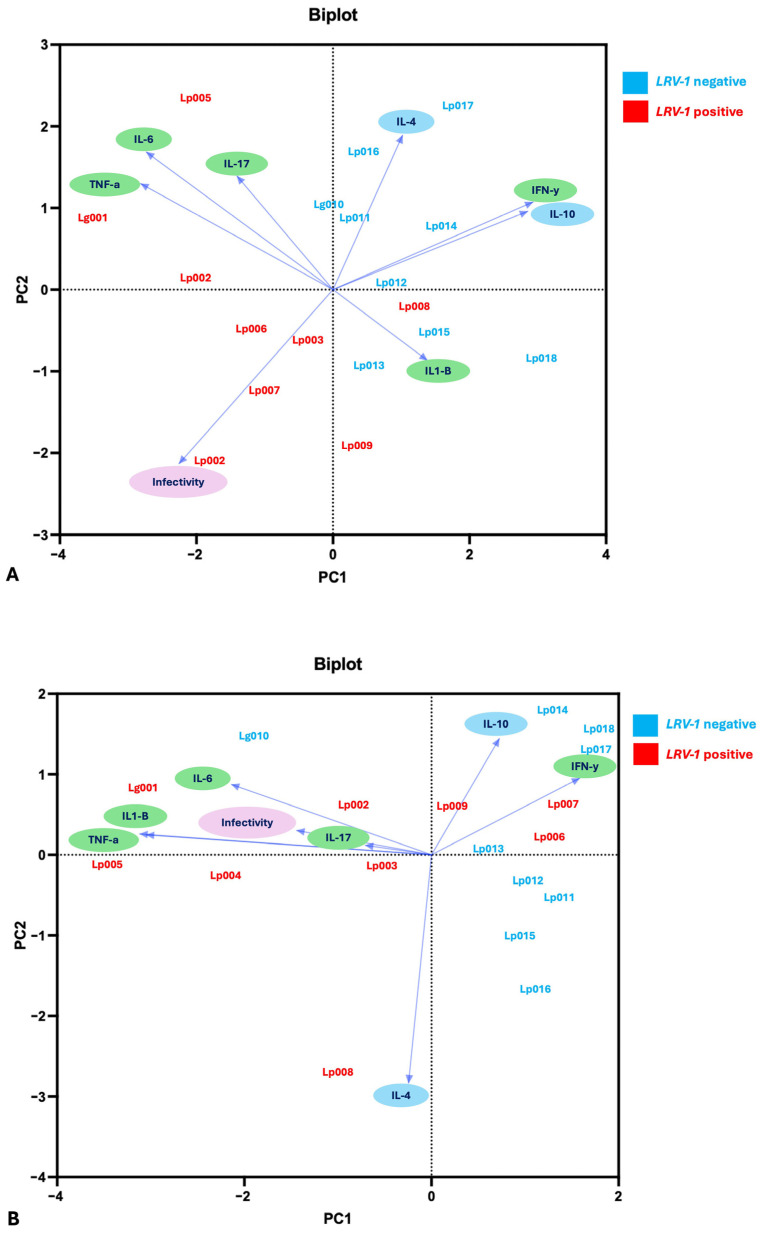
Biplot of the principal component analysis (PCA) showing the distribution of *Leishmania (Viannia) LRV-1*^+^ (red) and *LRV-1*^−^ (blue) isolates based on cytokine expression and infectivity index (II) variables at 24 and 48 h post-infection. 24 h (**A**). Proportion of explained variance for PC1: 29.47%, PC2: 20.63% and PC3: 14.19%. 48 h (**B**), Proportion of explained variance for PC1: 33.35%, PC2: 20.32% and PC3: 15.24%.

However, principal component analysis (PCA) using eight variables (infectivity, pro-inflammatory and anti-inflammatory cytokines), and the eighteen clinical isolates (nine *LRV-1*^+^ and nine *LRV-1*^−^) provided further insight into isolate clustering based on cytokine profiles (Figure 6).

At 24 h, *L. (Viannia) LRV-1*^−^ isolates were associated with IL-4, IL-10, and IFN-γ, whereas *LRV-1*^+^ isolates were more closely linked to IL-6, TNF-α, IL-17 (Lg001, Lp004, Lp005), and infectivity (Lp002 Lp006, Lp007, Lp003). IL-1β was shared across *LRV-1*^+^ (Lp008, Lp009) and several *LRV-1*^−^ isolates (Lp013, Lp015, Lp018) (Figure 6A).

After 48 h, PCA revealed a shift: pro-inflammatory cytokines (IL-6, IL-17, IL-1β, TNF-α) and II were primarily associated with *LRV-1*^+^ isolates (Lg001, Lp002, Lp003, Lp004, Lp005). Meanwhile, IL-10 and IFN-γ were linked to several *LRV-1*^−^ isolates (e.g., Lp011, Lp012, Lp013, Lp014, Lp017, Lp018), as well as to *LRV-1*^+^ isolates Lp006, Lp007, and Lp009 (Figure 6B).

## 4. Discussion

The role of *Leishmania RNA Virus 1* (*LRV-1*) in the biology and pathogenesis of *Leishmania* spp. remains incompletely understood. While studies in murine models have linked the presence of *LRV-1* to exacerbated cutaneous lesions [23], clinical data also suggest associations with treatment failure and symptomatic relapse in human leishmaniasis caused by species of the subgenus *Viannia* [15,16]. To explore the impact of *LRV-1* on parasite infectivity, we evaluated the infectivity index (II) of *Leishmania (Viannia)* isolates using U937 macrophage-like cells, a well-established in vitro model of human macrophage infection [29,46].

Consistent with previous findings [29], our results show that *LRV-1^+^* isolates exhibited significantly higher II than *LRV-1^−^* isolates, particularly after 48 and 72 h post-infection. This suggests that the presence of *LRV-1* may enhance parasite survival and intracellular replication within human macrophages, in line with the hypothesis that *LRV-1* acts as a virulence modulator through metabolic support or immune evasion [25,27,47].

When comparing *LRV-1^+^* isolates of *L. (V.) panamensis* and *L. (V.) guyanensis*, we observed a similar increase in II over time. This is particularly notable given that *L. (V.) panamensis* has historically been considered less virulent than other *Viannia* species [48,49,50]. Our findings suggest that *LRV-1* contributes to increased infectivity regardless of the intrinsic virulence traditionally attributed to the host species. Phylogenetic proximity between *L. (V.) panamensis* and *L. (V.) guyanensis* [51], as well as the circulation of the same *LRV-1* genotype (A) in both species [37], may explain their similar biological behavior in vitro.

Among the *LRV-1*^+^ isolates, two *L. (V.) panamensis* isolates (Lp004 and Lp005) showed the highest II values. These isolates originated from patients with more complex clinical courses: one with 11 concurrent lesions and the other requiring three treatment cycles with pentavalent antimonial. This observation suggests a potential link between high in vitro infectivity and complicated clinical manifestations. However, this association was not generalized as most *LRV-1*^+^ isolates (7/9) originated from patients with uncomplicated LCL. It is therefore plausible that other factors, such as parasite genotype or host immune status, contribute to disease severity [52,53]. The reported phylogenetic divergence of Panamanian *L. (V.) guyanensis* from South American strains [54], along with the presence of multiple *L. (V.) panamensis* haplotypes in Panama [55], may contribute to this intra-species variation in infectivity and clinical presentation.

Our cytokine analyses further support the immunomodulatory role of *LRV-1*. At 48 h post-infection, U937 macrophages infected with *LRV-1*^+^ isolates exhibited elevated concentrations of TNF-α and IL-1β and reduced IFN-γ, compared to those infected with *LRV-1*^−^ isolates. This cytokine profile is consistent with a pro-inflammatory profile that may promote parasite persistence by exacerbating local inflammation [16,23]. The reference strain Lg4147 (*LRV-1*^+^) exhibited similar cytokine behavior, reinforcing its value as a benchmark in in vitro models. Principal component analyses (PCA) at 48 h highlighted the association between the *LRV-1*^+^ isolates and increased TNF-α, IL-1β, IL-6, and II, supporting a coordinated inflammatory response. These results were reinforced with Spearman correlation analyses, where an association of IL-6 and TNF-α was observed in *LRV-1*+ isolates.

In contrast, *LRV-1*^−^ isolates were associated with a mixed cytokine profile, including higher levels of IFN-γ and IL-10. IFN-γ plays a key role in parasite clearance via nitric oxide-mediated macrophage activation [56]. Notably, isolates Lp004 and Lp005, which were associated with clinical complications, exhibited undetectable IFN-γ at 48 h, a finding that may explain their higher II and apparent survival advantage. The inverse relationship between IFN-γ and II further supports the notion that suppression of this cytokine facilitates infection in vitro.

Although no statistically significant differences were observed for IL-4, IL-6, and IL-10 between groups, we did observe increased IL-10 production at 48 h in both *LRV-1*^+^ and *LRV-1*^−^ isolates. This is consistent with previous studies suggesting that IL-10 contributes to parasite persistence by dampening host immune responses [57]. Interestingly, the *L. (V.) panamensis LRV-1*^−^ reference strain (LpS94) showed elevated IL-10 production, reinforcing its association with a regulatory immune profile described in cutaneous leishmaniasis [58].

Analysis of species-specific cytokine responses revealed that *L. (V.) guyanensis* isolates, both *LRV-1*^+^ and *LRV-1*^−^, tended to induce higher TNF-α levels, with Lg001 (*LRV-1*^+^) showing the highest concentrations at 48 h. Among *L. (V.) panamensis* isolates, Lp005 (*LRV-1*^+^) showed the highest TNF-α concentration, in agreement with its association with treatment failure. While TNF-α plays a key role in parasite killing, it is also implicated in tissue destruction during chronic inflammation [19,59,60]. Notably, IL-17 was detected in infections with both *LRV-1*^+^ and *LRV-1*^−^ isolates without significant inter-group differences. Given that macrophages are not primary producers of IL-17 [61,62] these results should be interpreted with caution, though this cytokine has been implicated in chronicity and parasite persistence in other models [47].

In this context, *LRV-1* detection could offer clinically relevant information by helping to identify patients at higher risk of persistent lesions or reduced treatment response, as suggested by other authors [15,16,43]. Due to the epidemiological importance of *L. (V.) panamensis* in Panama, adding *LRV-1* screening to diagnostic workflows or research surveillance systems might improve patient stratification and guide clinical decisions, especially in areas with high transmission rates [36]. Although more clinical and genomic research is necessary to confirm its predictive ability and to understand the effects of parasite genotype, host immunity, and environmental factors, including *LRV-1* as a supplementary biomarker, could be a significant step toward better disease management and public health strategies in endemic regions.

This study has limitations that should be considered when interpreting the findings. First, the limited number of isolates analyzed constrains the generalizability of the results, particularly regarding the intraspecific genetic diversity of *Leishmania (Viannia)* spp. and *LRV-1* genotypes, which nevertheless could, at least, be representative of dominant genotypes in Panama. Second, *LRV-1* detection was qualitative, and no quantification of viral load was performed using qRT-PCR. This limitation should be considered when interpreting the results, as the relative amount of virus could influence the degree of immunomodulation observed. Third, this study only used the U937 cell lineage. While the U937 in vitro model is a valuable tool for studying human macrophage infection, it does not fully replicate the complexity of the in vivo immune environment, which may influence the cytokine responses observed. Finally, the lack of longitudinal clinical or immunological data from the infected patients limits the ability to establish stronger correlations between *LRV-1* presence, immune response, and clinical disease progression.

Taken together, these findings support the hypothesis that *LRV-1* modulates host immune responses and enhances parasite infectivity, particularly in *L. (V.) panamensis* and *L. (V.) guyanensis* isolates. However, the presence of *LRV-1* alone does not fully explain clinical severity in cutaneous leishmaniasis cases in Panama. Most *LRV-1*^+^ isolates were associated with mild or moderate disease presentations, contrasting with reports from other regions where *LRV-1*^+^ has been linked to mucocutaneous leishmaniasis or treatment failure [5,6]

## 5. Conclusions

In conclusion, our findings demonstrate that *Leishmania (Viannia) LRV-1*^+^ isolates exhibit increased infectivity and are associated with a distinct pro-inflammatory cytokine profile characterized by elevated TNF-α and IL-1β and decreased IFN-γ, which may facilitate parasite survival in macrophages. Although the relationship between *LRV-1* and clinical outcomes is complex and likely influenced by parasite genetics, host immunity, and environmental context, our results indicate that *LRV-1* detection in *Leishmania (Viannia)* isolates from Panama could serve as a valuable prognostic marker for identifying tegumentary leishmaniasis cases with higher potential for persistence and therapeutic resistance. Given the predominance of *L. (V.) panamensis* in the country and the significant annual burden of cutaneous leishmaniasis, incorporating *LRV-1* screening into national surveillance programs and clinical management protocols could improve patient stratification, guide therapeutic decisions, and enhance disease control efforts in endemic regions of Panama. Further clinical and genomic studies are warranted to clarify the contribution of *LRV-1* to disease severity, drug responsiveness, and long-term parasite persistence in human populations.

## Figures and Tables

**Table 1:** General characteristics of the *Leishmania* isolates used in the in vitro infection experiments.

Isolate ID	*Leishmania* Species	*LRV-1 Status*	*Isolate Description*	Origin	Lesions
Lg001	*L. (V.) guyanensis*	*LRV-1* ^+^	Group 1	COC, PA	1
Lp002	*L. (V.) panamensis*	*LRV-1* ^+^	Group 1	DA, PA	1
Lp003	*L. (V.) panamensis*	*LRV-1* ^+^	Group 1	COL, PA	4
Lp004	*L. (V.) panamensis*	*LRV-1* ^+^	Group 1	PAC, PA	11
Lp005	*L. (V.) panamensis*	*LRV-1* ^+^	Group 1	COL, PA	1 ^^^
Lp006	*L. (V.) panamensis*	*LRV-1* ^+^	Group 1	PO, PA	1
Lp007	*L. (V.) panamensis*	*LRV-1* ^+^	Group 1	COL, PA	1
Lp008	*L. (V.) panamensis*	*LRV-1* ^+^	Group 1	BT, PA	1
Lp009	*L. (V.) panamensis*	*LRV-1* ^+^	Group 1	BT, PA	1
Lg010	*L. (V.) guyanensis*	*LRV-1* ^−^	Group 2	PO, PA	1
Lp011	*L. (V.) panamensis*	*LRV-1* ^−^	Group 2	COL, PA	1
Lp012	*L. (V.) panamensis*	*LRV-1* ^−^	Group 2	VE, PA	1
Lp013	*L. (V.) panamensis*	*LRV-1* ^−^	Group 2	PA, PA	1
Lp014	*L. (V.) panamensis*	*LRV-1* ^−^	Group 2	BT, PA	1
Lp015	*L. (V.) panamensis*	*LRV-1* ^−^	Group 2	DA, PA	2
Lp016	*L. (V.) panamensis*	*LRV-1* ^−^	Group 2	PA, PA	5
Lp017	*L. (V.) panamensis*	*LRV-1* ^−^	Group 2	PO, PA	2
Lp018	*L. (V.) panamensis*	*LRV-1* ^−^	Group 2	COL, PA	1
*Lg4147 (MHOM/BR/1975/WR4147)*	*L. (V.) guyanensis*	*LRV-1* ^+^	Reference strain	BR	N/A
*Lb566 (MHOM/BR/1975/M2903)*	*L. (V.) braziliensis*	*LRV-1* ^−^	Reference strain	BR	N/A
*LpS94 (MHOM/PA/1971/LS94)*	*L. (V.) panamensis*	*LRV-1* ^−^	Reference strain	PA	N/A

^^^ Treatment failure. Abbreviations: Lg = *L. (V.) guyanensis*; Lp = *L. (V.) panamensis*; Lb = *L. (V.) braziliensis*; BR = Brazil; PA = Panamá; PAC = Panamá province; COC = Coclé province; COL = Colón province; PO = Panamá Oeste province; BT = Bocas del Toro province; VE = Veraguas province; DA = Darién province. N/A: Not available.

## Data Availability

The data used to support the findings of this study are available from the corresponding author upon reasonable request, and the Supplementary Materials are available at: https://www.mdpi.com/article/10.3390/pathogens14121263/s1.

## Supplementary Materials

The following supporting information can be downloaded at: https://www.mdpi.com/article/10.3390/pathogens14121263/s1. Figure S1: Cytokine concentrations from *LRV-1*^+^ *Leishmania* (*Viannia*) isolates, as detailed in the accompanying table. Bar graphs (A,B) and spider web plots (C,D) represent cytokine profiles at 24 h (A,C) and 48 h (B,D) post-infection.; Figure S2: Cytokine concentrations from *LRV-1*^−^ *Leishmania* (*Viannia*) isolates, as detailed in the accompanying table. Bar graphs (A,B) and spider web plots (C,D) represent cytokine profiles at 24 h (A,C) and 48 h (B,D) post-infection.; Figure S3: Bar graphic showing the concentration of anti-inflammatory and pro-inflammatory cytokines of the reference strains, the LPS stimulation control, and the mean concentrations of the *LRV-1*+ and *LRV-1*- isolates used in this study. Figure S4: Heat map showing the results of correlation analysis between cytokines and infectivity in *LRV-1* positive and negative isolates at 24 h (A) and 48 h (B). A: Heat map showing r values (Spearman correlation) with statistical significance at 24 h: Moderate correlation directly proportional in *LRV-1*^+^ isolates: IL-6/TNF-α: r = 0.65, *p* = 0.03, and IL-6/IL-17: r = 0.60, *p* = 0.04; strong inversely proportional correlation in *LRV-1*^−^ isolates: IL-6/TNF-α: r = −0.73, *p* = 0.01. B. Heat map showing r values with statistical significance at 48 h: Strong proportional correlation in *LRV-1*^+^ isolates: IL-6/TNF-α: r = −0.79, *p* = 0.01.

## Abbreviations

The following abbreviations are used in this manuscript:

*LRV-1*	*Leishmaniavirus* type 1
*LRV-2*	*Leishmaniavirus* type 2
CL	Cutaneous Leishmaniasis
LCL	Localized Cutaneous Leishmaniasis
II	Infection Index
TNF-α	Tumoral necrosis factor alpha
IFN-γ	Interferon gamma
