# Supplementary figures and images for: Leishmaniavirus Type 1 Enhances In Vitro Infectivity and Modulates the Immune Response to Leishmania (Viannia) Isolates
Source: Pathogens. 2025 Dec 10;14(12):1263. doi: 10.3390/pathogens14121263 (PMC12735641; doi:10.3390/pathogens14121263)

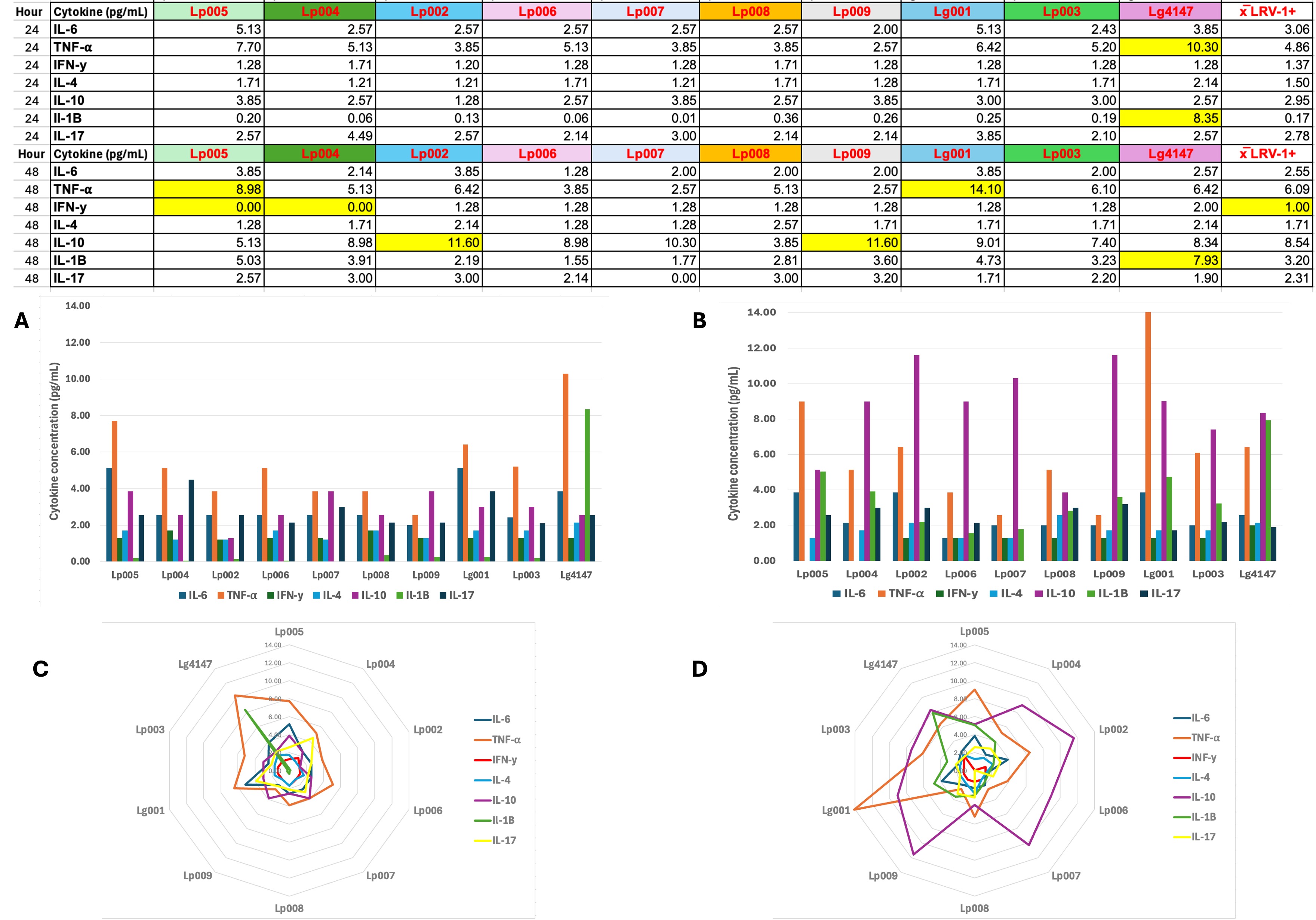

Supplement: Supplementary file 1 [file pathogens-14-01263-s001.zip › Figure S1.jpg]

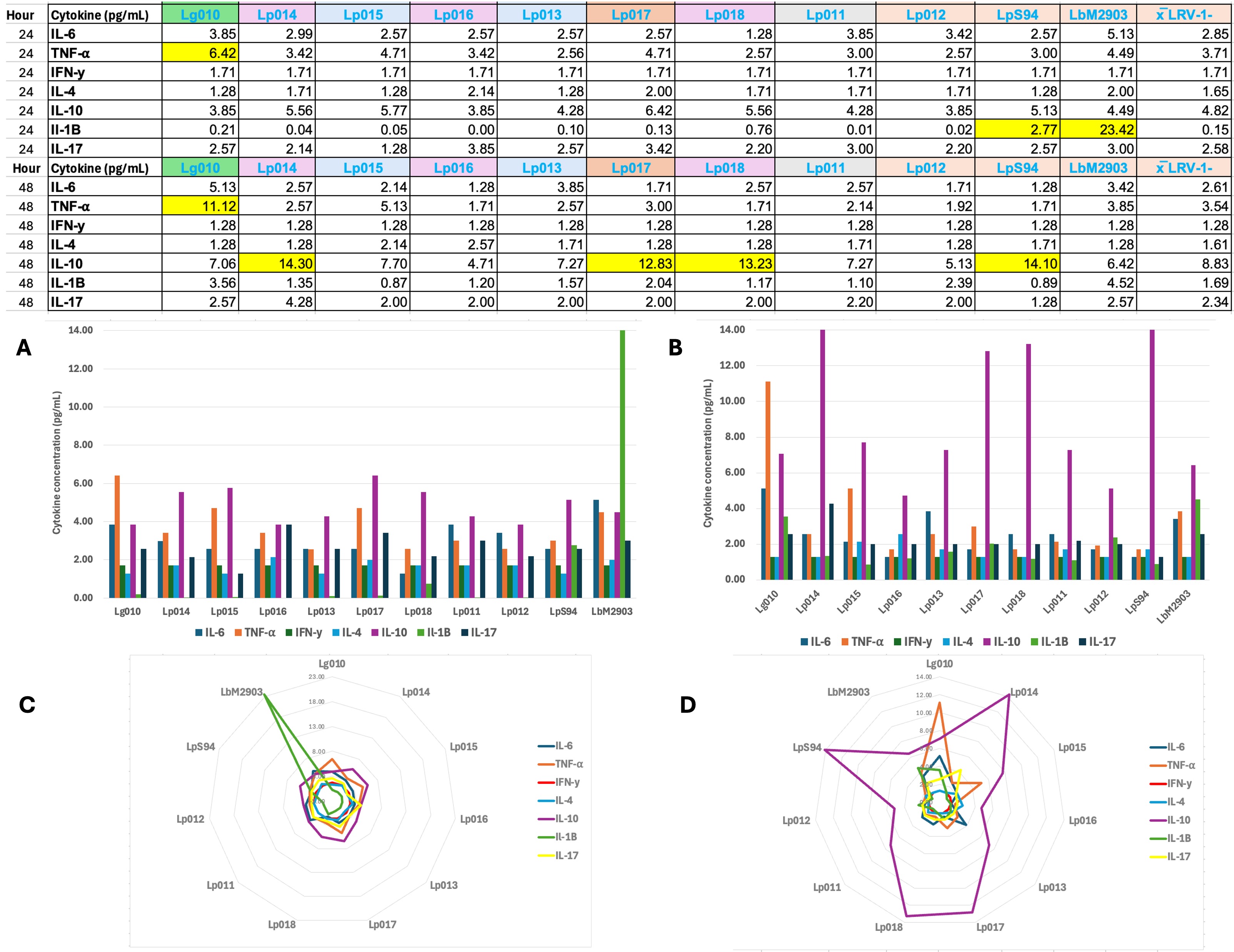

Supplement: Supplementary file 1 [file pathogens-14-01263-s001.zip › Figure S2.jpg]

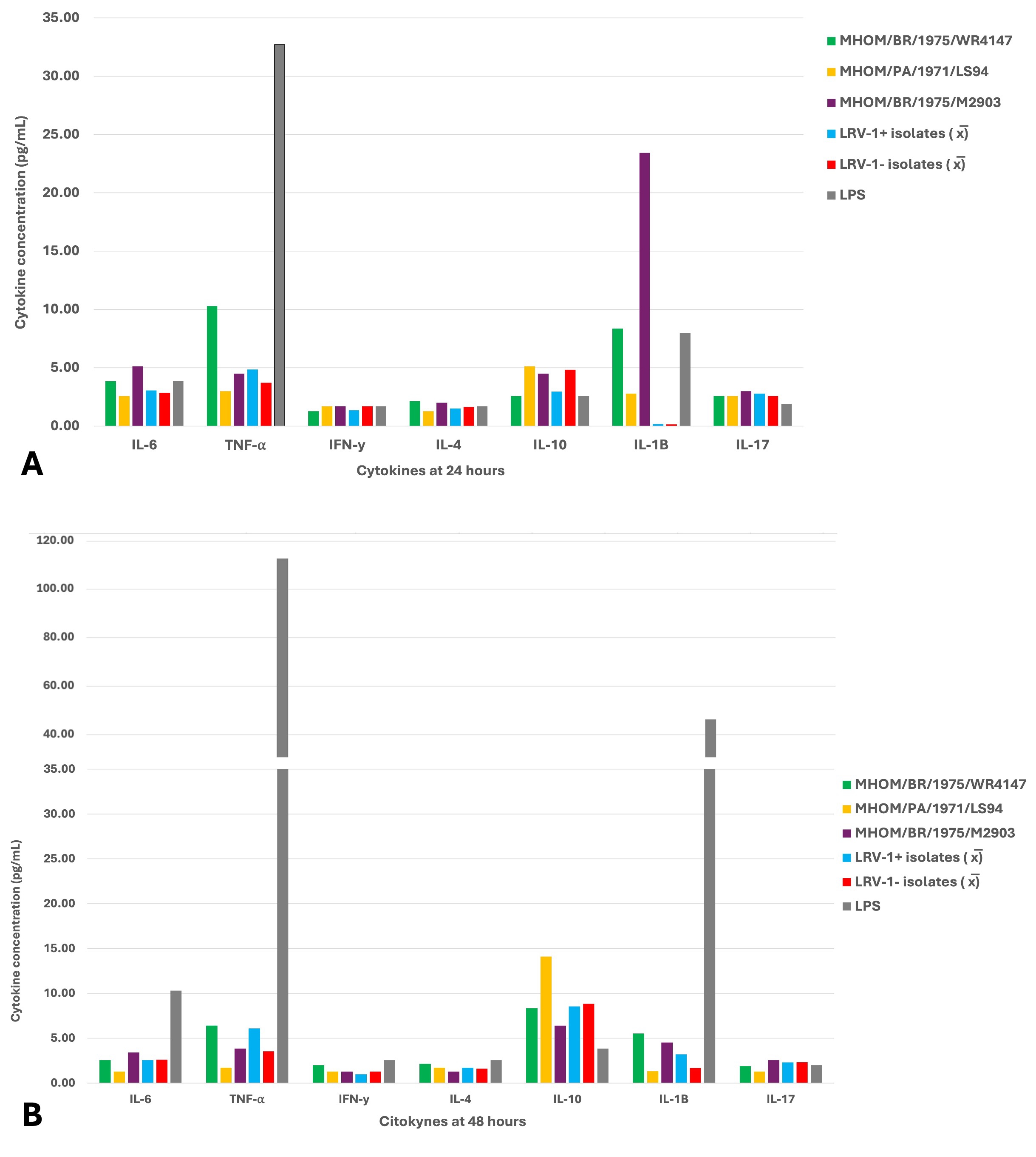

Supplement: Supplementary file 1 [file pathogens-14-01263-s001.zip › Figure S3.jpg]

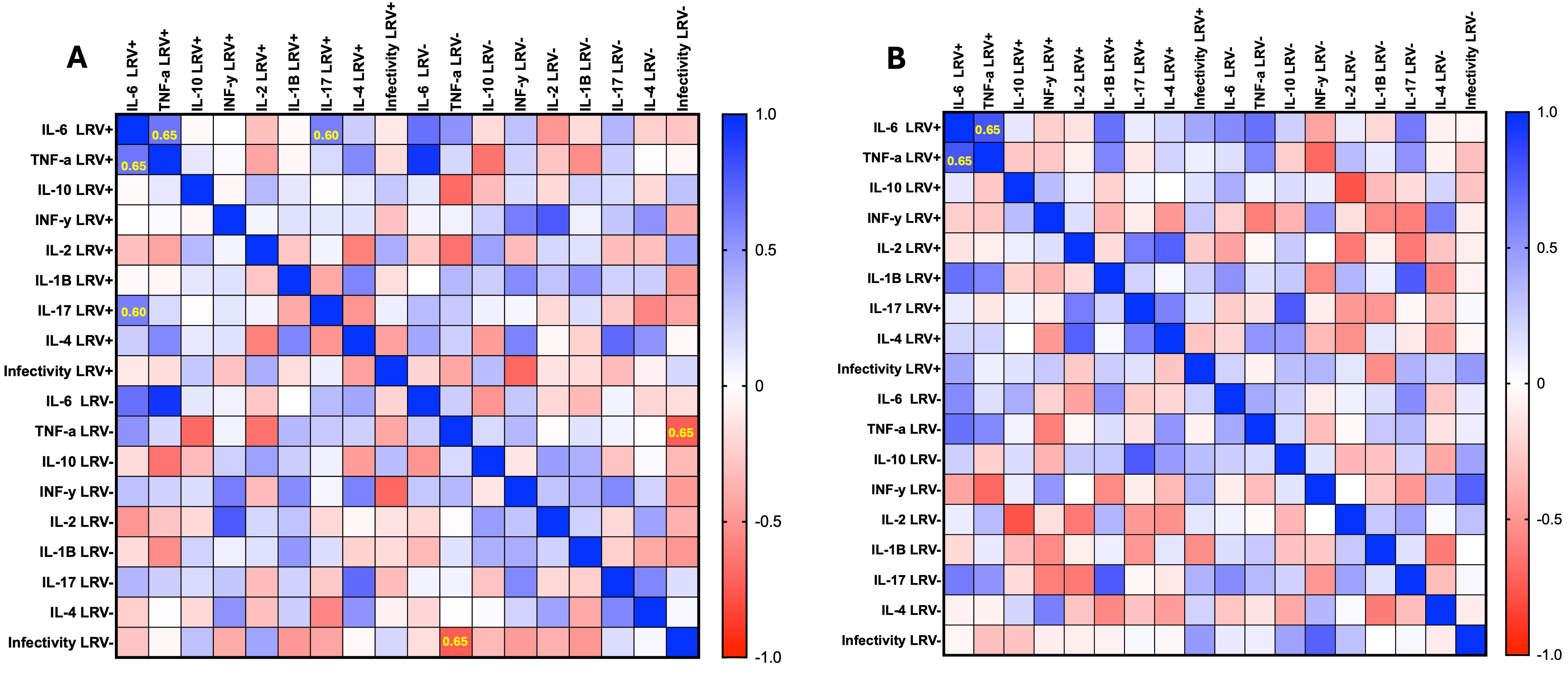

Supplement: Supplementary file 1 [file pathogens-14-01263-s001.zip › Figure S4.jpg]
